# Study on the compounding optimization of surfactants and synergistic effects on the wettability of bituminous coal

**DOI:** 10.1038/s41598-024-61266-1

**Published:** 2024-05-20

**Authors:** Deji Jing, Chunhua Bao, Zhe Dong, Xiangxi Meng, Xuefeng Han, Gang Li, Jingxu Chen

**Affiliations:** 1 College of Safety Science and Engineering, Liaoning Technical University, Fuxin, 123000 Liaoning China; 2 Key Laboratory of Mine Thermo-motive Disaster and Prevention, Ministry of Education, Fuxin, 123000 Liaoning China; 3 Erdos Research Insititute L.N Technical University, Ordos, 017000 Inner Mongolia China; 4 Sinosteel Maanshan General Institute of Mining Research Co.,Ltd., Maanshan, 243000 Anhui China; 5 College of Mining and Coal, Inner Mongolia University of Science and Technology, Baotou, 014010 Inner Mongolia China

**Keywords:** Surfactant, Compounding, Wettability, Quantum chemistry, Molecular dynamics simulation, Spray dust suppression, Health occupations, Engineering

## Abstract

To improve the wettability of surfactants on bituminous coal and to explore its wettability and wettability mechanism on bituminous coal, taking the Sandaogou bituminous coal as an example, a single factor experiment was carried out first. Through contact angle and surface tension experiments, three surfactants with good wettability were selected from among the nine surfactants and mixed in equal proportions two by two to determine the optimal compounding method and compounding concentration. The experimental results show that the compounding of nonionic and anionic, nonionic and zwitterionic, anionic and zwitterionic surfactants can have synergistic effects and significantly improve the wettability of bituminous coal. Among them, the 0.5 wt% SDS + 0.5 wt% CAB-50 (R2) compound surfactant had the best wettability on bituminous coal, and the contact angle and surface tension were only 15.24° and 23.62 mN/m, respectively. The surface electrostatic potential values of each material molecule were calculated by Materials Studio software based on the quantum chemistry method, and correlation analysis was carried out with wettability. The results show that the surface electrostatic potentials of CDEA, SDS and CAB-50 were greater than those of water and bituminous coal, and the region of maximum negative electrostatic potential corresponded to oxygen atoms, which are easier to adsorb on bituminous coal and water molecules. Then, through molecular dynamics simulation, the interaction energy and the distribution of contributions along the Z-axis of the water/compound surfactant/bituminous coal system at equilibrium were investigated, and finally, a spray dust reduction test was carried out in the Sandaogou Coal Mine. The results showed that the 0.5 wt% SDS + 0.5 wt% CAB-50 compound solution can be used as a water molecule adsorption carrier, prompting more water molecules to be embedded into coal molecules, increasing the relative concentration of water molecules on the surface of bituminous coal, restricting the diffusion of water molecules, and greatly improving the wettability. After the addition of 0.5 wt% SDS + 0.5 wt% CAB-50 as a spray agent, the concentration of total dust at the driver's position decreased from 65.14 to 9.11 mg/m^3^, the concentration of exhaled dust decreased from 30.07 to 3.35 mg/m^3^, and the efficiency of total and exhaled dust reduction compared with that of pure water was 86.01% and 89.35%, respectively.

## Introduction

As the main energy source in China, coal accounts for approximately 70% of the country’s energy supply^1^. During coal mining, transport and processing, a large amount of respirable particles are generated^2^, which not only accelerate the abrasion and ageing of production equipment and trigger major accidents such as coal dust explosions but also seriously threaten the health of coal miners^3^. At the end of 2021, a total of 15,407 cases of various types of occupational diseases were reported nationwide, including 11,809 cases of occupational pneumoconiosis^4^. The situation of occupational disease prevention and control in China is still severe^5,6^, therefore, how to efficiently reduce dust has always been an urgent problem for coal mining enterprises in China.

At present, coal mine underground currently commonly used dust removal methods are ventilation, wet, physicochemical and dry. Ventilation dust removal is widely used because of its simple equipment, but the dust removal efficiency is relatively low. Although physicochemical dust removal technology has high dust removal efficiency, but whole dust removal process is relatively complex. Although dry dust removal does not consume water, its overall equipment is bulky, inconvenient to operate, and occupies a large space^7–9^. Spray dust reduction technology is widely used in underground coal mines due to the advantages of simple overall equipment, convenient operation, high dust reduction efficiency, etc.^8,10^. Due to the high hydrophobicity of dust and high surface tension of pure water, the capture efficiency of ordinary water mist on dust is not ideal^11–13^. Surfactant can reduce the surface tension of water. With the addition of surfactant, the solution will gradually reach micelle concentration value, during this process, the force of the liquid surface of the liquid is gradually decreases, which is conducive to spreading and wetting of the liquid at solid interface, to improve the wettability^14,15^. Currently, many scholars have carried out much research on surfactants affecting coal wettability, the remarkable progress has been made in theory, experiments, and field applications^16^. Xi et al.^17^using scanning electron microscopy, studied the wettability and adhesion of different surfactants, observed that surfactant solution has excellent wetting ability and greater adhesion than pure water. Niu et al.^18^found surfactant can reduce the surface tension of pure water and pure water contact angle on coal mine, significantly improve the wettability of solution and coal dust. Meng et al.^19^studied the wettability of four anionic surfactants by surface tension and contact angle experiments, and found that SDBS had the lowest surface tension and superior wettability. Zhang^20^ found through sedimentation experiments that AEO-9 had a sedimentation rate of only 1 mg/s for coal dust with a particle size in the range of 0.05–0.061 mm.

In summary, how to improve the wettability of water and coal is an important factor to inhibit the diffusion of dust release, and the wetting effect is related to coal type. At present, but there have been fewer studies on wetting effect of surfactants on bituminous coal^21,22^. Beside, most studies on wettability mechanism of different surfactants are limited to experimental demonstration and macroscopic analysis, which largely restricts the further development of coal seam water injection and spray dust removal technology^23,24^. However, due to the extremely complex coal structure, exploring wettability mechanism can theoretically prove the influencing factors of coal wettability, effectively improve wettability, accurately improve the dust reduction efficiency^25,26^. Therefore, this study took Sandaogou bituminous coal as research object, evaluated the influence of different surfactants on wettability of bituminous coal by contact angle and surface tension. The optimized monomeric surfactants were compounded to determine the optimal compound method and optimal surfactant concentration suitable for Sandaogou bituminous coal. Quantum chemical simulation and molecular dynamics simulation were used to reveal the mechanism of wetting bituminous coal by the compound surfactants at microscopic level. The contact process between the compound surfactants and bituminous coal were simulated through the establishment of water/compound surfactant/coal system to analyse interaction relationship between the system from perspective of surface electrostatic potential, the interaction energy and the distribution of contributions along the Z-axis, and to compare the simulation results with experimental results to demonstrate that. Finally, the spray dust reduction test was carried out on site. The results of the study can provide a reference for study of surfactants wetting of bituminous coal.

## Experiment and simulation and test

### Preparation and properties of the coal sample

**Figure 1:**
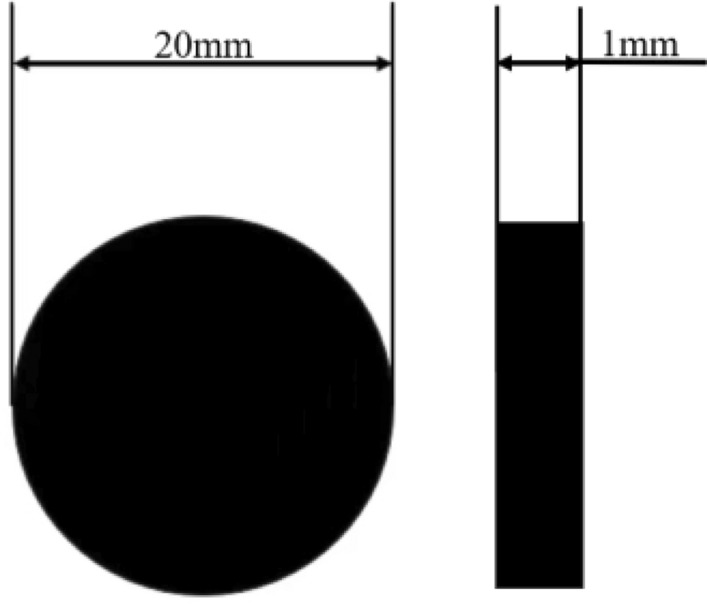
Experimental coal sample.

Note: ‘Mad’ denotes the moisture content of the air-dried base material, ‘Aad’ denotes the ash content of the air-dried base material, and ‘Vad’ denotes the volatile content of the air-dried base material, and ‘Vdaf’ denotes the volatile matter of the air-dried base material on an ash free basis.

The experimental coal sample was prepared from bituminous coal of fully mechanized 45,205 working face of the Sandaogou Coal Mine. The coal sample was crushed by a pulveriser and screened to obtain 150 mesh coal powder, which was pressed into a cylindrical shape using a YP-40 tablet press at 30 MPa, as shown in Fig. 1. The coal sample was analysed according to the GB/T30732-2014 "Coal Industrial Analysis Methods Instrumental Method".

### Preparation of surfactant compound solutions

Nine types of surfactants^27,28^, which are nontoxic, harmless, have good solubilization and penetration potential, were selected from the market. The nine surfactants were prepared in solutions with mass concentrations of 0.001 wt%, 0.005 wt%, 0.01 wt%, 0.05 wt%, 0.1 wt%, and 0.5 wt%. Through preliminary experiments, according to the effect of wetting bituminous coal with different concentrations of surfactants, reagents referred to as CDEA, SDS, and CAB-50 were screened. The optimal monomeric surfactant solutions were mixed two by two in equal proportions to obtain compound solutions. The parameters of each surfactant are shown in Table 1.

**Table 1:** Types of surfactants.

Species	Abbreviation	Chemical name of surfactants	Chemical formula
Nonionic	AEO-9	Primary alcohol Ethoxylate	C_30_H_62_O_10_
	CDEA	Coconut diethanol Amide	C_11_H_23_CON(CH_2_CH_2_OH)_2_
	DNPEO	Alkylphenol ethoxylates	C_19_H_32_O_3_
Anion	SDS	Sodium dodecyl sulfate	C_12_H_25_SO_4_Na
	SDBS	Sodium dodecyl benzene sulfonate	C_18_H_29_NaO_3_S
	MES	Fatty acid methyl estersulfonate	C_6_H_13_NO_4_S
Amphoteric ion	DTAB	Dodecyl trimethyl ammonium bromide	C_15_H_34_N·Br
	CAB-50	Cocoamidopropyl betaine	C_19_H_38_N_2_O_3_
	BS-12	Amphiprotic surfactant BS-12	C_24_H_45_N_1_O_2_

### Experimental methods

1) Determination of surface tension.

The surface tensions of the nine surfactant monomeric solutions and three compound solutions were determined by the hanging slice method with a ZL-500A surface tension metre; the surface tension of each solution was determined three times, and the average value was taken.

2) Determination of solutions contact angle on coal sample.

The contact angles of the nine surfactant monomeric solutions and three compound solutions on the coal sample were measured by droplet method using a ZJ-7000 contact angle measuring instrument. Specifically, after droplet is placed on coal sample, the test is started immediately, the test is stopped after droplet is completely adsorbed into coal dust tablet, the program is used to calculate average contact angle. The experiments were repeated three times for each group, and taken average value.

### Quantum chemical simulation

**Figure 2:**
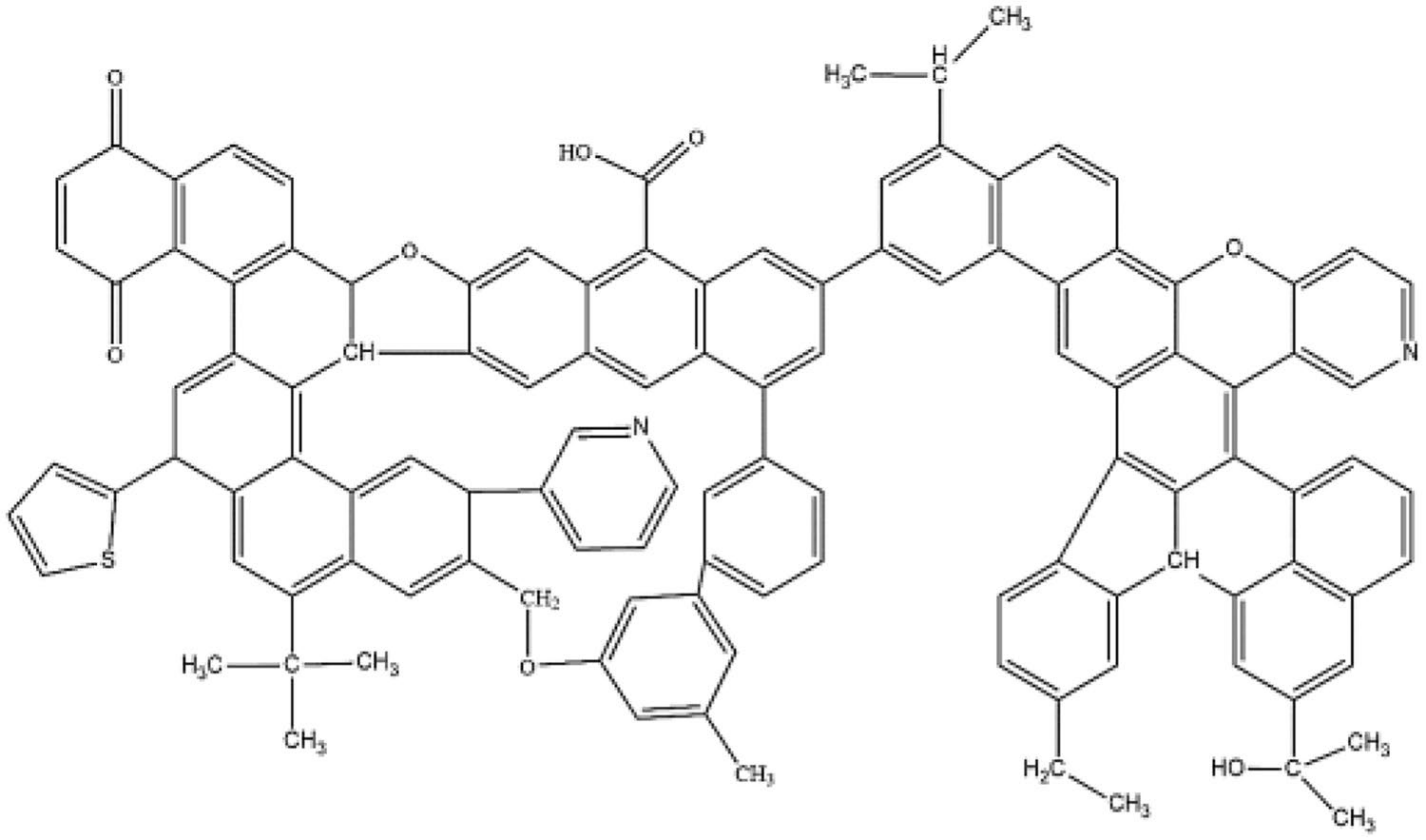
Plane model of the molecular structure of the coal sample.

The quantum chemistry calculation module DMol^3^ in Materials Studio software was used to geometrically optimize the simplified models of coal, water and nine surfactant molecules, and the electrostatic potential of each molecular surface was calculated. The adsorption of water in bituminous coal molecular model was judged by the electrostatic potential of related molecular surface, then the wettability of water in bituminous coal molecules was judged. Similarly, the adsorption law of different surfactants on surface of bituminous coal molecules were also be determined. The team continuously adjusted the element position and optimized the structure of the constructed model and obtained the molecular structure plane model of bituminous coal, as shown in Fig. 2. Its molecular formula is C_116_H_82_O_8_N_2_S.

**Figure 3:**
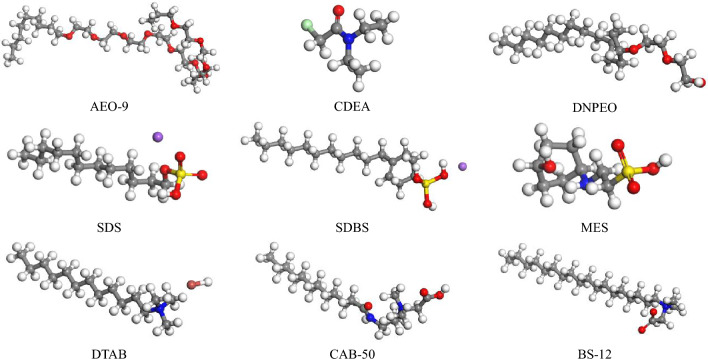
Molecular models of surfactants of different types.

The molecular formula and structural formula of surfactants were determined by the CAS number, then a molecular model of surfactants was constructed and continuously optimized with Materials Studio software. The models of 9 surfactants were obtained as shown in Fig. 3.

### Molecular dynamics simulation

**Figure 4:**
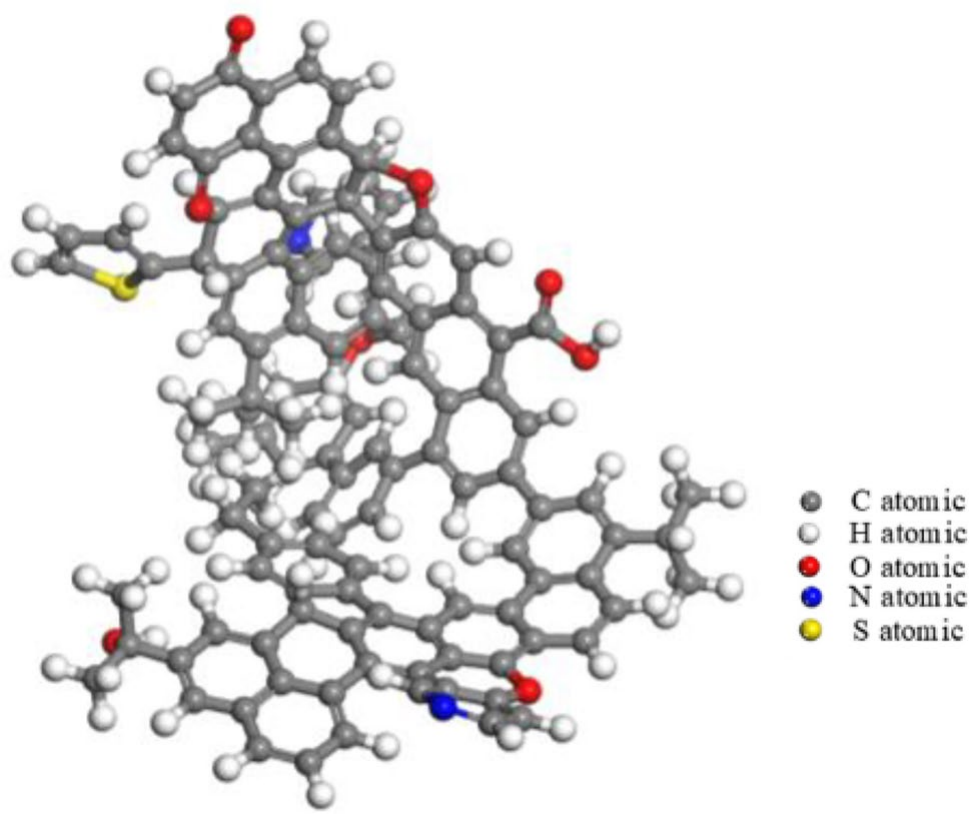
The lowest energy configuration of coal molecule.

**Figure 5:**
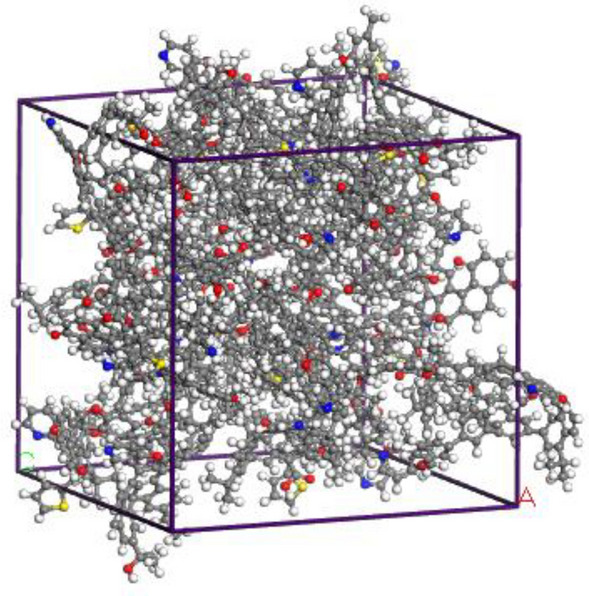
Aggregation model of coal sample.

Geometry optimization was carried out using geometry optimization under the Forcite module, with the mass accuracy was set to Fine, simulation step number was set to 100,000, force field optimized COMPASSII, with simulated charge assignment selected Forcefield assigned, to obtain the lowest energy configuration (see Fig. 4). Then, 20 optimized anthracite molecules were randomly added to the cell with dimensions of 36.4 Å × 36.4 Å × 36.4 Å by Amorphous Cell module, and three-dimensional periodic boundary conditions were added. The density of the structural model was set to 1.3 g/cm^3^. After geometry optimization, the samples were annealed using high-temperature relaxation, followed by NVT system synthesis and the Nose temperature-controlled method. The initial temperature was set to 300 K, the maximum temperature was set to 800 K, the heating rate was 50 K/time, and the simulation time was 200 ps. The aggregation state model of the bituminous coal molecules in Sandaogou coal seam shown in Fig. 5 was obtained.

**Figure 6:**
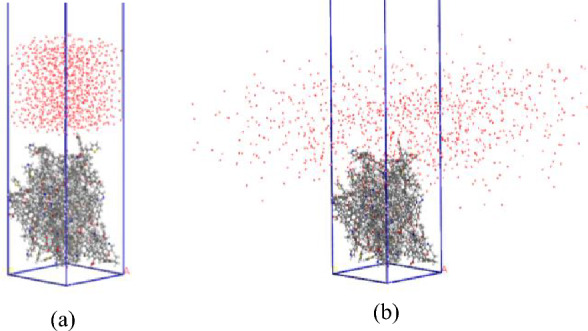
Initial configuration and Dynamic simulation diagram of coal/water system. (**a**) Initial configuration (**b**) Dynamic simulation diagram

The coal molecular aggregation structure model was subsequently added to the constructed three-dimensional unit model, a vacuum layer with thickness of 100 Å was added to the top of coal surface, to avoid interactions between the top surface and the bottom surface of model due to periodic boundary conditions. The Amorphous Cell module was used to construct a container containing 800 water molecules, and used Build Layer tool to add water molecular layer to coal surface to construct a coal/water interface adsorption model, as shown in Fig. 6.

**Figure 7:**
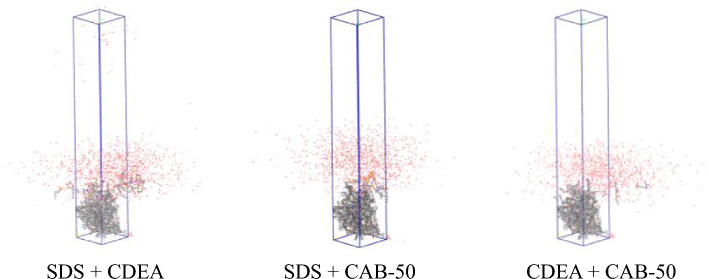
Dynamic simulation of the coal/compound surfactant/water system.

Finally, three compound systems were constructed by using the AC module in Materials Studio software: 10 SDS molecules and 10 CDEA molecules, 10 SDS and 10 CAB-50 molecules, 10 CDEA and 10 CAB-50 molecules. The three groups of molecules were put into the constructed model, the remaining parameters were consistent with those stated above, as shown in Fig. 7.

### Test method

Pure water, 0.5 wt% SDS, 0.5 wt% CAB-50, and 0.5 wt% SDS + 0.5 wt% CAB-50 were used as test reagents in spray dust reduction test, along with a LG-120 self-developed nozzle was used. Zhang Tian et al.^29^. conducted a series of studies on nozzles. Liu Hongwei et al.^30^ applied the nozzle to implement full-section combined dust control technology in the return air crossheading of the 01 fully mechanized mining face in Mindong No.1 Coal Mine. The dust concentration was significantly reduced, but the technical device was not combined with the compound surfactant to study dust reduction. Therefore, this study combines the equipment with a compound surfactant to carry out a dust reduction test.

According to the field investigation, the existing pressure air flow and pressure situation of 45,205 fully mechanized mining face in Sandaogou support opening of 10 frames and 30 nozzles at the same time. The atomization pressure of each single nozzle was set to 0.2–0.5 MPa, the gas consumption was 5.5 m^3^/h, and the water consumption was 168 mL/min. Combined with water consumption of a single nozzle, the total water consumption of operating system was 5 L/min. According to the highest solubility and ratio (5/1000), the volume of a single liquid storage tank was 17 L, 1700 g of active agent was added at one time.

An AKFC-92A dust sampler was used to monitor the driver's movement during sampling. The sampling point was set within range of breathing zone at the driver's location. The sampling flow rate was 20 L/min, the sampling duration was 10 min. The sampling port was relative to the direction of airflow and deviated from the working surface by 30° to ensure the accuracy of sampling data. The filter membrane was weighed before and after sampling on a balance, these weights were recorded. By measuring the weight change of filter membrane before and after sampling, the mass of dust in a unit volume of air can be obtained. The working conditions were measured three times, after which the average dust concentration was calculated and recorded.

Next, the collected dust-containing filter membrane was dried and weighed, the concentration of total dust in the air was calculated according to Formula 1:

1 $$\rho { = }\frac{{m_{2} - m_{1} }}{Q \times t} \times 1000$$

In the formula:

where* ρ* is the dust mass concentration. mg/m^3^;

*m*_1_ is the total mass of the filter membrane before sampling. mg;

*m*_2_ is the total mass of the dust-containing filter membrane after sampling. mg;

*Q* is the sampling flow rate. L/min;

*t* is the sampling time. Min.

By recording the change in mass concentration under different working conditions, the dust reduction efficiency under each working condition was calculated. The calculation formula for the dust reduction efficiency is as follows:

2 $$\eta = \frac{{\rho_{{2}} - \rho_{{1}} }}{{\rho_{{2}} }} \times 100\%$$

In the formula:

*η* is the average dust removal efficiency. %;

*ρ*_1_ is the average dust concentration after dust fall. mg/m^3^; and.

*ρ*_2_ is the average dust concentration before dust fall. mg/m^3^.

## Results and discussion

### Analysis of the experimental results

The results of industrial analyses are shown in Table 2

**Table 2:** Industrial analysis of the experimental coal sample.

Mad (%)	Aad (%)	Vad (%)	Vdaf (%)
0.82	7.32	21.56	23.89

**Figure 8:**
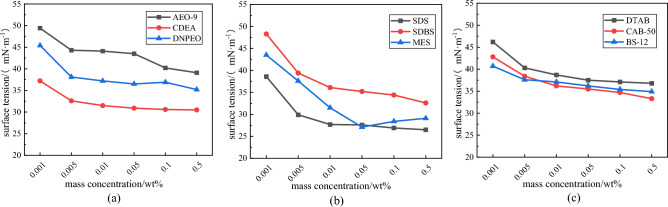
Surface tension of various types of surfactants at different mass concentrations. (**a**) Nonionic surfactants (**b**) Anionic surfactants (**c**) Amphoteric surfactants

Surface tension and contact angle are important parameters for determining the wetting ability of solution on coal body; the lower the surface tension and contact angle are, the easier the solution breaks up and soaks into coal dust, the better the solution wetting properties are. The surface tension test results are shown in Fig. 8. Under the experimental conditions, the surface tension of pure water is 71.97 mN/m.

In Fig. 8, the surface tension values of nonionic solutions are separated by concentration of 0.005 wt%, at which point the slope of line decreases sharply and then changes slowly, during which the surface tension of CDEA reaches a minimum value of 30.5 mN/m at concentration of 0.5 wt%. The surface tension values of anionic solutions first decrease sharply, when the concentration reached 0.05 wt%, SDS and SDBS changed gradually and tended to stabilize, while MES appeared to grow in the opposite direction, this because solution concentration reached the critical micelle concentration (CMC), a large number of micelle groups formed in solution, and hydrophobic groups in the solution of the gas/liquid interface rows reached a saturated state. At this point, increasing the concentration did not reduce surface tension, and the reverse effect may have occurred. The surface tension of SDS in anionic solution at concentration of 0.5 wt% was only 26.5 mN/m. The surface tension of amphoteric ion solutions first decreased sharply and then slowed down, with CAB-50 reaching a minimum of 33.3 mN/m at a concentration of 0.5 wt%.

**Figure 9:**
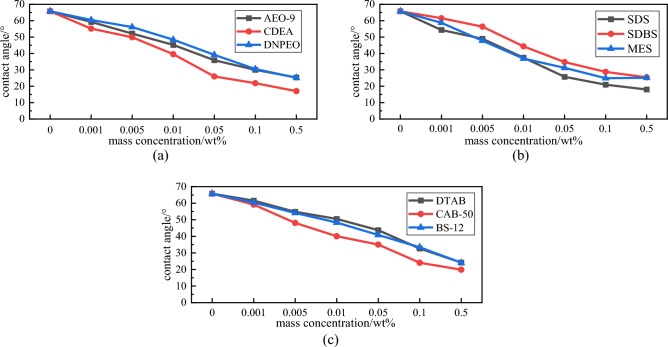
Contact angles on coal mine of various types surfactants at different mass concentrations. (**a**) Nonionic surfactants (**b**) Anionic surfactants (**c**) Amphoteric surfactants

The contact angle results for each surfactant on coal sample are shown in Fig. 9. The pure water contact angle on the coal sample is 65.75°.

The decreasing trends of contact angles for all nine surfactants are relatively flat when surfactant mass concentration is increased to 0.01 wt%. After concentration is greater than 0.01 wt%, the surface activity of solutions increase, the contact area with the coal sample increases, the solutions easily spread on the surface of coal sample, the contact angles start to decrease dramatically. When the mass concentration reaches 0.5 wt%, the nonionic solutions contact angles on the coal sample decreased by an average of 65.76% compared with that pure water on the coal sample, of which the contact angle of CDEA decreased by as much as 74.11%, which indicates better wettability. Compared with that of pure water on coal sample, the anionic solutions contact angle on the coal sample decreased by 65.24% on average. The decrease rate of the SDS contact angles on the coal sample were as high as 72.62%, the wettability property was better than those of the remaining two samples. The amphoteric ionic solutions contact angles on coal sample decreased by 65.46% on average compared with that of pure water on coal sample, among which the contact angle of CAB-50 decreased by 69.79% to only 19.86°, which was the lowest among the three amphoteric ionic surfactants.

The compound solutions were numbered in order as R1 (0.5 wt% SDS + 0.5 wt% CDEA), R2 (0.5 wt% SDS + 0.5 wt% CAB-50), and R3 (0.5 wt% CDEA + 0.5 wt% CAB-50). The results of the experiments of the compound surfactants are shown in Table 3.

**Table 3:** Contact angle and surface tension of each solution.

Solution	Contact angle with coal sample/°	Surface tension/(mN m^−1^)
R1	17.89	28.45
R2	15.24	23.61
R3	18.14	28.94
0.5 wt% SDS	18.83	26.58
0.5 wt% CDEA	17.02	30.51
0.5 wt% CAB-50	19.86	33.39

As shown in Table 3, the contact angles of R2 after compounding on coal sample were 19.07% and 23.26% lower than the contact angles at the same concentration of the two monomers, 76.82% lower than the contact angle of pure water. This is because dipole-ion interactions may be generated between the polar head groups of the two surfactants, which reduces the electrostatic repulsion between surfactants. In addition, the hydrophobic effect of molecular hydrocarbon chain facilitates the formation of micelles in compound solution, reduces the critical micelle concentration (CMC) and surface tension of compound solution, improves the surface activity, and increases the ease of adsorption by coal dust. The surface tension of 0.5 wt% SDS is the lowest among the nine monomer surfactants, at only 26.58 mN/m. The surface tension of R2 solution is reduced by 11.17% and 29.29% compared with that of 0.5 wt% SDS solution and 0.5 wt% CAB-50, respectively. This is because of the strong electrostatic interaction between anionic and amphoteric ionic surfactants, which greatly reduces the absorption free energy of each component in mixed system, improves the surface activity of the compounding system.

### Analysis of simulation results

1) Electrostatic potential analysis.

The electrostatic potential (ESP) refers to the work required to move a unit of positive charge from infinity to this point. The electrostatic potential of molecular surface can predict the site of electrophilic reaction and the site of the nucleophilic reaction, determine the strength of hydrogen bond formed between two molecules, determine the magnitude of intermolecular interaction energy, and analyse the wettability.

**Figure 10:**
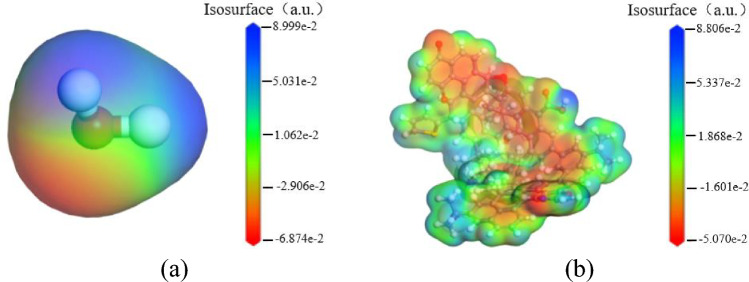
Surface electrostatic potential distribution of water and coal sample. (**a**) Water (**b**) Coal sampie

**Figure 11:**
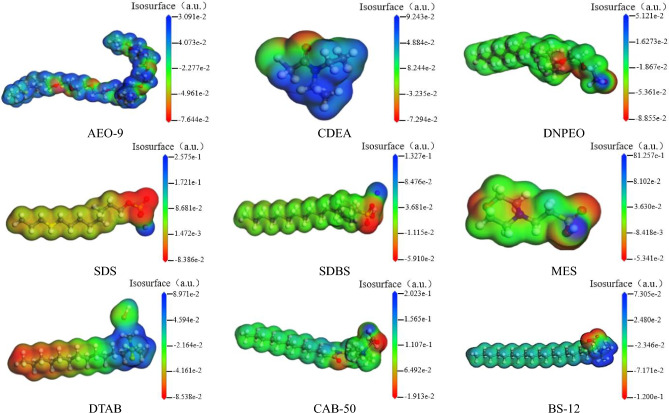
Electrostatic potential distribution of different surfactants.

The calculated electrostatic potential distribution of each molecule surface is shown in Fig. 10, Fig. 11. Red represents negative potential, which provides electrons for the formation of hydrogen bonds; blue represents positive potential, which mainly accepts electrons to form hydrogen bonds; green represents potential that is close to zero and relatively stable structure. In Fig. 10, the positive area of electrostatic potential on surface of water molecules is mainly distributed at positions of hydrogen atoms, and the negative area is mainly located near oxygen atom. The functional group corresponding to the maximum electrostatic potential of bituminous coal surface is oxygen atom of carboxyl group, the functional group corresponding to the minimum value is the position of phenolic hydroxyl hydrogen atom. The distribution range of electrostatic potential fluctuates less, the extreme points are mainly attributed to oxygen-containing functional groups, pyridine and pyrrole. In addition, the surface electrostatic potential of bituminous coal is lower than that of water; that is, the electrostatic force between water molecules and bituminous coal is less than that between water molecules and water molecules, indicating that the wettability of the Sandaogou bituminous coal is poor and that it is not easy wetted.

Table 4 shows the maximum values of electrostatic potential on surface of each molecule. The maximum positive potential of water molecule is 0.0923 a.u., the maximum negative potential is − 0.06698 a.u. Normally, the positive and negative potentials within water molecule are attracted to each other through hydrogen bonding. When the electrostatic potential of surfactant is greater than that of water molecule, the electrostatic force between surfactant molecules and water molecules is greater than the electrostatic force between water molecules is, the surfactant molecules are more likely to form hydrogen bonds with water molecules, thereby adsorbing and wetting. As shown in Table 4, the surface electrostatic potentials of CDEA, SDS, CAB-50 are greater than those of the coal sample and water, which easily produces hydrogen bonds with coal and water, the maximum negative potentials of the surface electrostatic potentials of the three surfactants are located near the oxygen atoms, which more easily combine with bituminous coal and water molecules. Additionally, these surfactants play a bridging role in the three-phase system of coal/surfactant/water, which further enhances wettability.

**Table 4:** The maximum electrostatic potential of the different molecules.

Molecule name	Negative potential/a. u	Positive potential/a. u
Water	− 0.06983	0.09236
Coal sample	− 0.05070	0.08806
AEO-9	− 0.07645	0.03091
CDEA	− 0.07294	0.08942
DNPEO	− 0.08855	0.05121
SDS	− 0.08386	0.25748
SDBS	− 0.05910	0.13271
MES	− 0.05314	0.12574
DTAB	− 0.08238	0.08971
CAB-50	0.01913	0.20229
BS-12	− 0.11997	0.07305

2) Interaction energy analysis.

By calculating the interaction energy of the three contact surfaces of water/compound surfactant/coal surface, the magnitude of the system interaction strength can be judged, and thus, the effect of surfactant on coal wettability can be determined. The lower the energy is, the greater the negative interaction energy is, which means that adsorption between water molecules and coal is more stable and more conducive to wetting. The calculation formula of the interaction energy between the systems is as follows:

3 $$E_{i} = E_{t} - (E_{c} + E_{s}+ E_{w})$$

where *E*_*i*_ is the interaction energy between surfactant and coal, kJ/mol;

*E*_*t*_ is the total energy of system, kJ/mol;

*E*_*c*_, *E*_*s*_ and *E*_*w*_ are the energy of coal, surfactant and water, respectively, kJ/mol. The energy of the other three is calculated by removing the other two in system.

Table 5 shows that the energy changes in system result in negative binding energies, indicating the reactions between water molecules and bituminous coal molecules are spontaneous. A larger negative value indicates the reaction is more spontaneous and thorough. In contrast, a lower negative value proves the reaction is more unstable. The order of absolute values of interaction energy in coal sample is SDS + CAB-50 > CDEA + CAB-50 > SDS + CDEA, indicating the combination of anionic and amphoteric ion surfactants in coal sample has the strongest ability to bind water. Consistent with the experimental results, this shows the compound surfactant can effectively increase the contact between water and coal, thereby improving its wetting ability.

**Table 5:** Energy changes in the coal sample system.

System	E_non_	E_van_	E_ele_	E_tol_	E_int_
SDS + CDEA	− 10,821.90	2375.27	− 13,197.17	− 23,476.85	− 2081.22
SDS + CAB-50	− 14,181.99	2529.92	− 16,711.91	− 29,782.67	− 3343.04
CDEA + CAB-50	− 13,087.96	2339.79	− 15,427.75	− 24,765.27	− 2204.85

3) Radial distribution analysis along Z axis.

**Figure 12:**
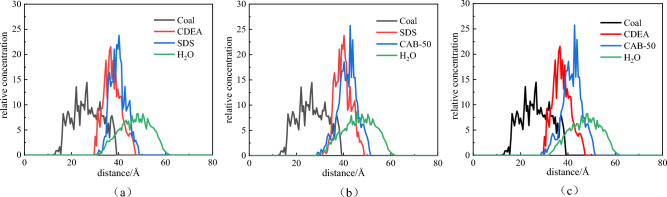
Distribution of the three-phase system in bituminous coal along the Z-axis. (**a**) SDS + CDEA (**b**) SDS + CAB-50 (**c**) CDEA + CAB-50

The radial distribution along Z-axis refers to the ratio of number density of a certain particle in slice of simulated system to total number density of particles in system in Z-axis direction. By analysing relative concentration distribution of system along Z-axis direction, the effect of surfactants on interface between bituminous coal and water can be elucidated. To further analyse the adsorption capacity of compound surfactant on surface of bituminous coal, according to the results of molecular dynamics simulation, the contribution distribution of each component along Z-axis in three-phase system was calculated, the role of the compound surfactant at interface between bituminous coal and water was clarified. The distribution curves of the different systems are shown in Fig. 12.

As shown in Fig. 12, since the position of bituminous coal is fixed, the relative concentration distribution ranges in systems of three compound solutions are basically same, and are mainly concentrated in range of 15 Å to 38 Å. In the three-phase system, the addition of compound solution promoted the embedding of more water molecules in coal sample and compound solution. It is obvious after adding the SDS + CAB-50 solution, the number of water molecules in coal, SDS molecules and CAB-50 molecules increases, and the relative concentration of water molecules on surface of bituminous coal increases, that is, the SDS + CAB-50 solution facilitates the adsorption and wettability of water molecules on surface of bituminous coal.

**Figure 13:**
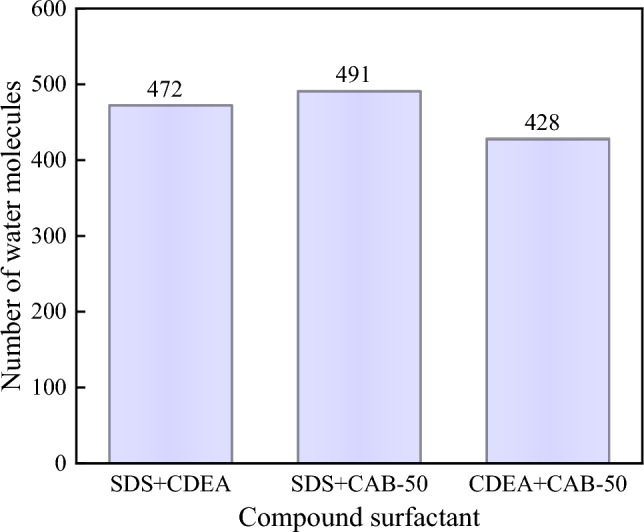
Number of water molecules in the bituminous coal system with different compound surfactants.

To explore the contact between water molecules and coal sample more accurately, the data of water molecules infiltrating inside and on the surface of coal molecules were extracted and collated. As shown in Fig. 13, there are 472 water molecules when SDS + CDEA is added, 491 when SDS + CAB-50 is added, and 428 when CDEA + CAB-50 is added. The addition of SDS + CAB-50 compound solution to coal sample system promoted the movement of more water molecules to coal, and the wettability of water molecules on surface of bituminous coal increased, which proves that this solution has the best wetting effect on coal sample.

### Test results analysis

The measured filter membrane was weighed and calculated to obtain total dust concentration, and the dispersion degree was analysed by filter membrane dissolution smear method to obtain the exhaled dust concentration. In this study, the dust concentrations under four working conditions were obtained, and the results are shown in Table 6.

**Table 6:** Mass concentration changes in total dust and exhaled dust at the driver's location under different working conditions.

Spraying method	Total dust concentration/(mg m^−3^)	Exhaled dust concentration/(mg m^−3^)
Pure water	66.13	29.76
	65.26	30.51
	65.02	29.94
0.5 wt% SDS	16.75	7.89
	16.52	8.96
	16.81	8.68
0.5 wt%CAB-50	18.64	10.04
	18.82	10.83
	19.25	9.67
0.5 wt% SDS + 0.5 wt% CAB-50	9.36	3.02
	9.21	3.22
	8.76	3.35

Table 6 shows that after adding pure water for spray dust reduction, the average total dust concentration was 65.14 mg/m^3^, the average exhaled dust concentration was 30.07 mg/m^3^. After adding 0.5 wt% SDS, the average total dust concentration was 16.69 mg/m^3^, the average exhaled dust concentration was 8.51 mg/m^3^, the efficiency of dust reduction compared with that of pure water was 74.38% of total dust, 71.70% of exhaled dust, after adding 0.5 wt% CAB-50, the average total dust concentration was 18.90 mg/m^3^, the average exhaled dust concentration was 10.18 mg/m^3^, the efficiency of dust reduction compared with that of pure water was 74.38%, 71.70%, respectively. After addition of 0.5 wt% CAB-50, the average total dust concentration was 18.90 mg/m^3^, the average exhaled dust concentration was 10.18 mg/m^3^, the dust reduction efficiency was 70.99% for total dust, 66.15% for exhaled dust. After adding 0.5 wt% SDS + 0.5 wt% CAB-50, the total dust concentration decreased from 65.14 to 9.11 mg/m^3^, the dust reduction efficiency reached 86.01%, the exhaled dust concentration decreased from 30.07 mg/m^3^ to 3.35 mg/m^3^, the exhaled dust reduction efficiency reached 89.35%, compared to 0.5 wt% SDS with better monomer effect, the total dust and exhaled dust improved by 11.63, 17.65 percentage points, respectively. Compared with that of ordinary water mist, the dust reduction efficiency of compound surfactant greatly improved, the dust reduction effect on exhaled dust was greater, minimizing the small-size coal dust deposited deep into respiratory tract, proving that 0.5 wt% SDS + 0.5 wt%CAB-50 greatly improved the solution's ability to wet coal dust when it was used as water for spraying.

## Conclusion

1. Surface tension and contact angle experiments showed that the three monomer surfactants, CDEA, SDS and CAB-50, had high wettability. After compounding, 0.5% wt% SDS + 0.5% wt% CAB-50 compound surfactant was the best solution for Sandougou bituminous coal, which showed superior synergistic effect with contact angle of only 15.24° and surface tension of only 23.62 mN/m.
2. The surface electrostatic potential results showed that the surface electrostatic potentials of CDEA, SDS and CAB-50 were greater than those of water and bituminous coal, and the region with the maximum negative value of their electrostatic potentials corresponded to the oxygen atoms, which made it easier to adsorb bituminous coal and water molecules; additionally, these materials bridged the three-phase system of coal/surfactant/water, which greatly improved the wetting effect.
3. The joint analysis of the interaction energy of the system and the distribution of contributions along the z-axis based on molecular dynamics simulations showed that: After adding 0.5 wt% SDS + 0.5 wt% CAB-50 solution, the relative concentration of water molecules on the coal surface increased and the adsorption was more stable, the water molecules were more likely to aggregate on the coal surface; the interaction energy in the coal/0.5 wt% SDS + 0.5 wt% CAB-50/water system can be increased, showed that the incorporation of 0.5 wt% SDS + 0.5 wt% CAB-50 molecules restricts the diffusion of water molecules, implied that adsorption is more likely to occur, the wettability is enhanced. It can be useful for the validation of performance of new surfactants for wetting coal dust.
4. The spray dust reduction test showed that after adding the compounded surfactant, the concentration of total dust at the driver's position decreased from 65.14 to 9.11 mg/m^3^, the concentration of exhaled dust decreased from 30.07 to 3.35 mg/m^3^, and the efficiency of dust reduction compared with that of pure water was as high as 86.01% and 89.35% for total dust and exhaled dust, respectively. This effectively improves the wettability of the bituminous coal and greatly improves the underground working environment of Sandougou Coal Mine.

### Supplementary Information

Supplementary Information.

## Data Availability

All data generated or analysed during this study are original and are included in this published article.
