# Supplementary material for: Study on the compounding optimization of surfactants and synergistic effects on the wettability of bituminous coal
Source: Sci Rep. 2024 May 20;14:11461. doi: 10.1038/s41598-024-61266-1 (PMC11106322; doi:10.1038/s41598-024-61266-1)

Supplementary information

1、The details of the 9 surfactants.

Full name of surfactants	structural formula	source	Advantages and properties
Primary alcohol Ethoxylate	R-O-$(CH_2CH_2O)_nH$(R=C12~18,n=15~16)	Guangzhou Gao Yue Chemical Co	Good emulsifying and dispersing properties; hydrophilic emulsifier, which can enhance the solubility of certain substances in water.
Coconut diethanol Amide	RCON（CH_2CH_2OH）$_2$	Guangzhou Chita Import & Export Co	It has significant thickening, foam-enhancing and foam-stabilising properties; it has good compatibility and synergistic effect with other surfactants.
Alkylphenol ethoxylates	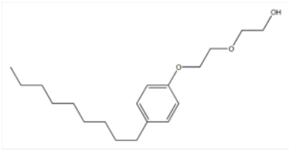	Tianjin Hiens Biochemical Technology Co	Excellent detergency, wetting and emulsifying properties.
Sodium dodecyl sulfate	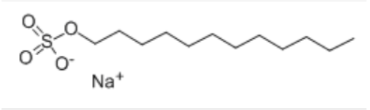	Hunan Yongqi Chemical Co	White to slightly yellow powder, slightly special odour, easily soluble in water, can be used as raw materials, modification of materials.
Sodium dodecyl benzene sulfonate	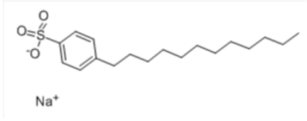	Shanghai Hongshun Biotechnology Co	Colour or light yellow powdery or flaky solid. Difficult to volatilise, easily soluble in water, dissolved in water into translucent solution.
Fatty acid methyl estersulfonate	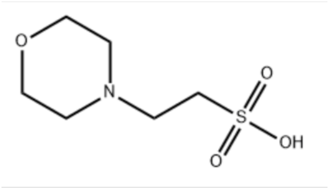	Nanjing Quest Biotechnology Co	White solid powder at room temperature and pressure, soluble in water, ethanol, methanol, acetone and other polar solvents,

			no obvious toxicity and irritation to organisms.
Dodecyl trimethyl ammonium bromide	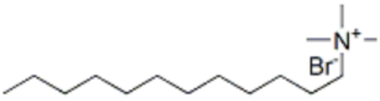	Hunan Huibaiyi New Material Co	White to slightly yellow, used as emulsifier for natural and synthetic rubber and asphalt, disinfectant for silkworms, antistatic agent for synthetic fibre, fungicide for oil field water injection.
Cocoamidopropyl betaine	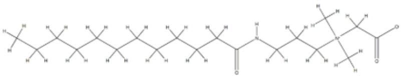	Shandong Ammi Chemical Technology Co	In combination with other surfactants, it has a good synergistic effect and can reduce the irritation caused by traditional surfactants
Amphiprotic surfactant BS-12	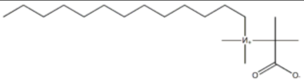	Zhengzhou Ruike Biochemical Technology Co	Colourless or light yellow transparent liquid with excellent foaming properties

2、The image of parameter setting (operation interface of the procedures) of using Forcite module to simulate are as follows:

(1) Geometry optimization was carried out using geometry optimization under the Forcite module.

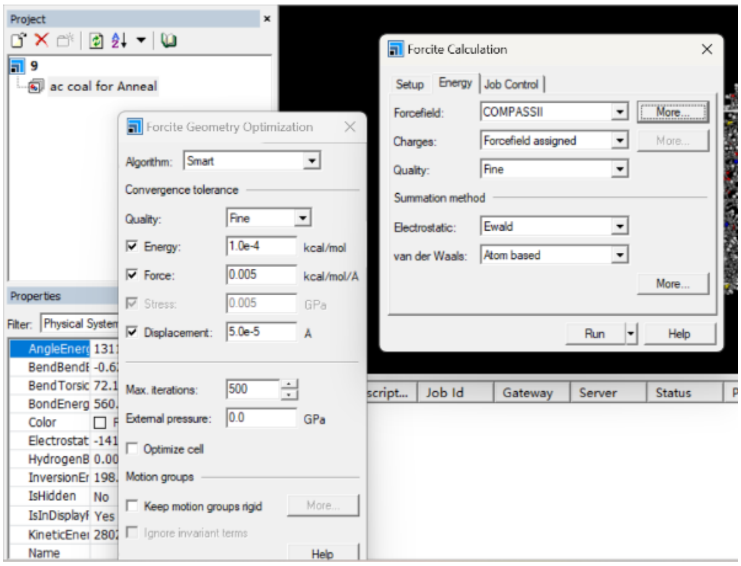

(2) The samples were annealed using high-temperature relaxation.

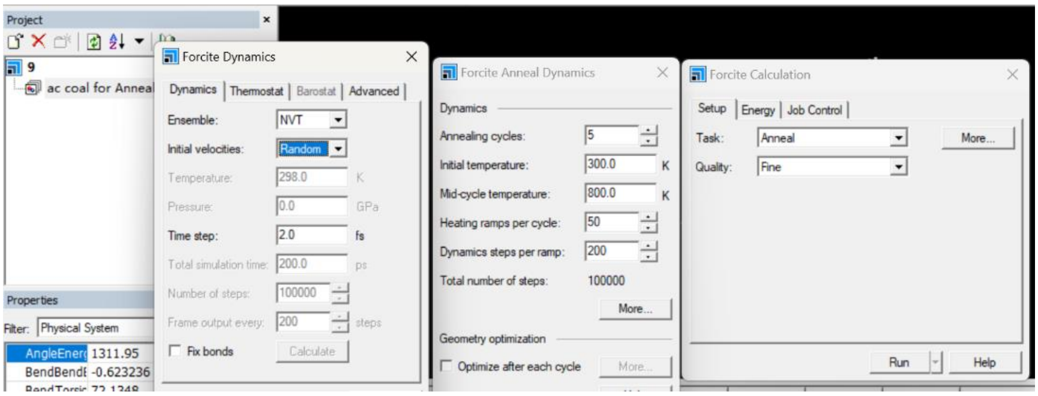

(3) A vacuum layer with a thickness of 100 Å was added to the top of the coal surface.

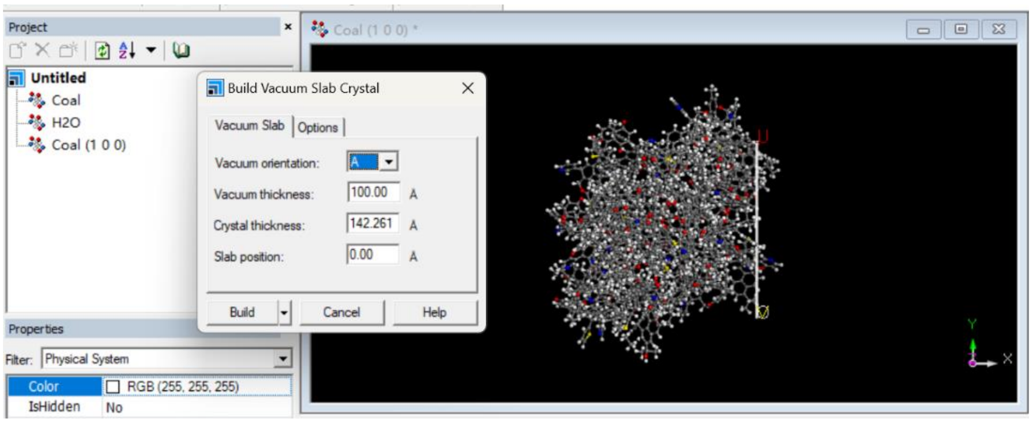

(4) To construct a coal/water interface adsorption model.

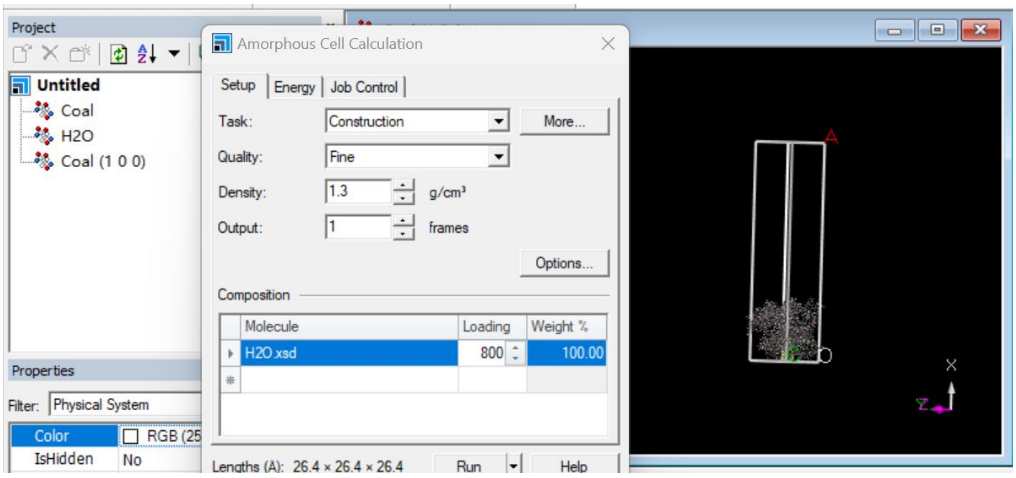

(5) To construct the coal/compound surfactant/water system.

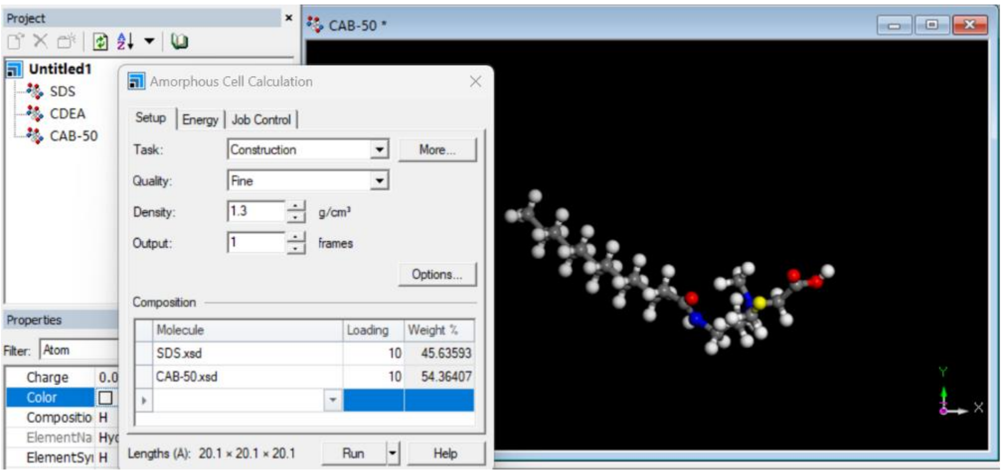

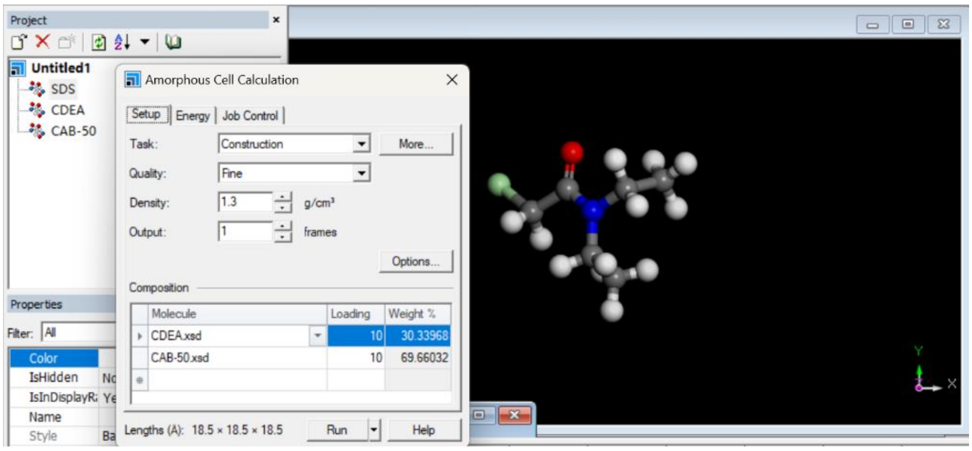

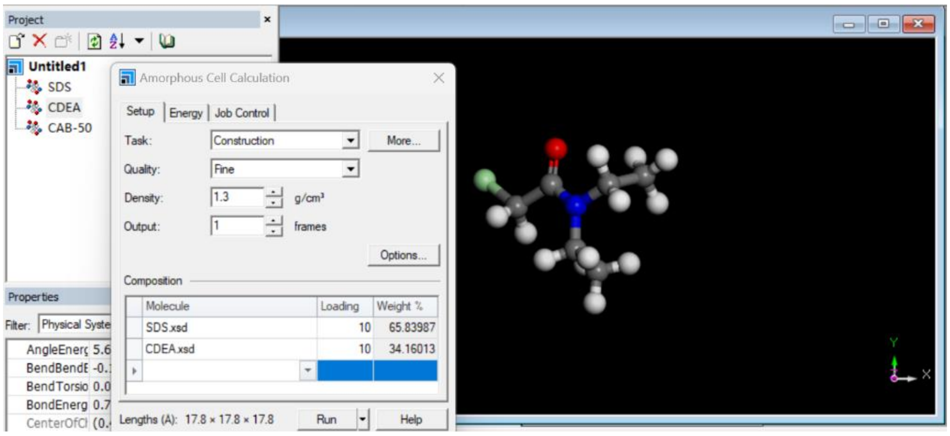

3、The photos of the new equipment setting in field application are as follows:

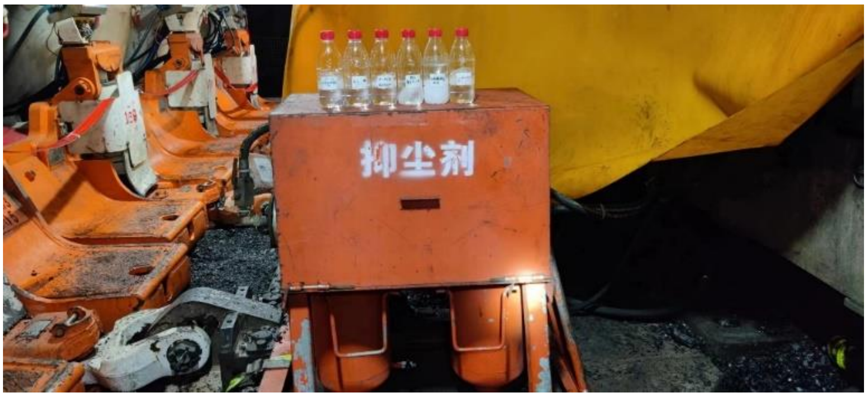

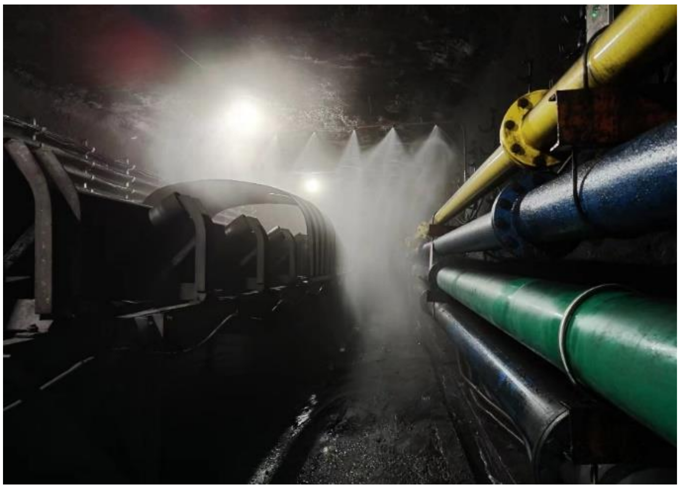

4、ZL-500A tensiometer:

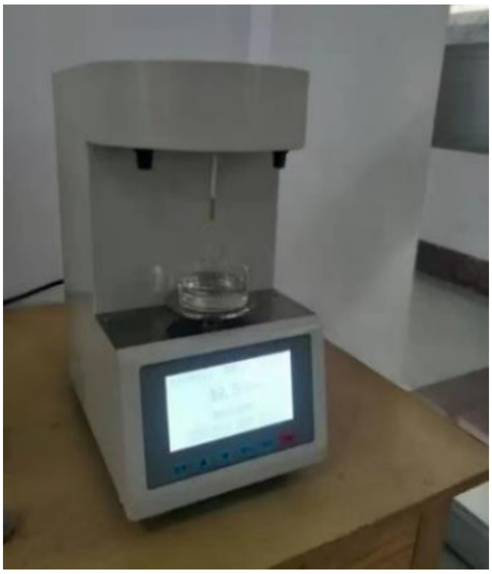

5、ZJ-7000 optical contact angle measuring instrument:

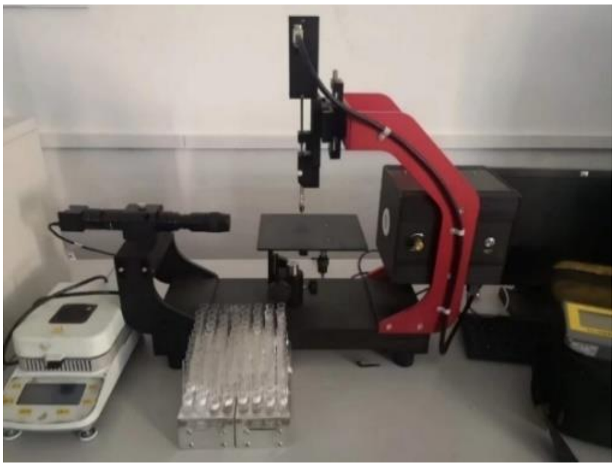

Supplement: Supplementary file 1 — Supplementary Information. [file 41598_2024_61266_MOESM1_ESM.pdf]
